# Secondary peritoneal leiomyomatosis after uterine leiomyoma intervention: multi-institutional clinical and radiologic experience

**DOI:** 10.1007/s00261-026-05637-4

**Published:** 2026-06-26

**Authors:** Leslie W Nelson, Mark D Sugi, Christine O Menias, Katharina F Feister, Vincent M Mellnick, Lisa M Ho, Grace G Zhu, Perry J Pickhardt

**Affiliations:** 1 Department of Radiology, University of Wisconsin–Madison, Madison, USA; 2 Department of Radiology, Mayo Clinic, Scottsdale, USA; 3 Department of Radiology, Mallinckrodt Institute of Radiology, Washington University Medical Center, St Louis, USA; 4 Department of Radiology, Duke University, Durham, USA; 5 Department of Radiology, University of Utah, Salt Lake City, USA

**Keywords:** Peritoneal leiomyomatosis, Secondary peritoneal leiomyomatosis, US, CT, MRI

## Abstract

**Purpose:**

To assess the clinical and imaging features of secondary peritoneal leiomyomatosis (SPL) in women with a history of uterine leiomyoma intervention.

**Methods:**

Multi-institutional review identified 31 women with peritoneal leiomyomatosis, including 28 cases of SPL related to prior uterine leiomyoma intervention (mean age at index CT, 42.7 ± 10.6 years). Patient demographics, surgical history, imaging (US/CT/MR/PET), and pathology were reviewed. Retrospective imaging review of the peritoneal findings was performed by board-certified abdominal radiologists.

**Results:**

Of the 28 female patients with SPL, 12 underwent prior myomectomy and 16 underwent prior hysterectomy for primary uterine leiomyoma treatment. In 17 cases, power morcellator use during the procedure was confirmed. SPL was diagnosed an average 7.2 ± 4.5 years after primary fibroid surgery, with 78.6% (22/28) being symptomatic. Mean size of the largest peritoneal-based lesion on index CT was 8.0 ± 5.6 cm, with the number of lesions ranging from 1 to too numerous to count (TNTC) (mean, 4.1 ± 3.8 lesions, excluding 2 TNTC cases). Typical US/CT/MR findings included multiple rounded/lobulated, enhancing peritoneal-based nodules/masses with imaging characteristics similar to uterine leiomyomata. 24 patients underwent tumor resection/debulking surgery, and one patient underwent CT-guided biopsy. Available pathology results included benign leiomyomas (*n* = 22) and leiomyosarcoma (*n* = 3). Five patients went on to develop additional documented SPL recurrence following initial resection an average 6.5 years later (range, 0.7–16.0 years).

**Conclusion:**

This study highlights a rare, mostly benign and symptomatic, complication following surgical intervention of intrauterine leiomyomas. Despite the literature focus on morcellator-related peritoneal leiomyosarcoma dissemination, only a small minority of our cases proved to be malignant.

## Introduction

 Uterine leiomyomas (also known as uterine fibroids) are the most common pelvic tumors in women, with an estimated prevalence ranging from 20 to 77% depending on age, ethnicity, and diagnostic technique [1–3]. Although these are benign lesions, up to 30% of patients may experience a wide range of symptoms, including pain, pressure, abnormal uterine bleeding, and infertility [4, 5]. Treatment options for symptomatic patients include hysterectomy, myomectomy, hormonal therapy, and embolization. Myomectomy is a surgical technique used to remove uterine fibroids while preserving the uterus via laparoscopic or open surgical methods [1, 5]. If the leiomyoma is too large for specimen extraction via the port site, intracorporeal morcellation can be performed to fragment the large specimen for piecemeal retrieval. This technique has been widely accepted and utilized in laparoscopic myomectomies and hysterectomies [6–8]. However, an increased risk for subsequent peritoneal dissemination has been documented, which has necessitated the use of a tissue containment system [9].

Disseminated peritoneal leiomyomatosis (also known as leiomyomatosis peritonealis disseminata) is a rare, mostly benign condition of multiple extra-uterine leiomyomas within the peritoneal cavity. Historically, the etiology of these lesions was thought to originate from peritoneal metaplasia or from pedunculated subserosal myomas that torse and parasitize. However, as noted above, an iatrogenic cause after myomectomy or hysterectomy for treatment of leiomyomas has emerged as a compelling pathogenesis, especially with morcellator use [10]. When patients are found to have developed peritoneal leiomyomatosis after primary uterine leiomyoma intervention, this is specifically referred to as secondary peritoneal leiomyomatosis (SPL).

While SPL is a relatively rare complication following primary leiomyoma intervention, it should be considered if an appropriate patient presents with multiple peritoneal-based soft tissue nodules and masses of varying sizes. To date, the medical literature has focused primarily on the even rarer complication of peritoneal leiomyosarcomas following the use of power morcellators [11–14]. Consequently, we believe that the more common benign complication (i.e., SPL) is under-recognized and the initial misdiagnosis rate is extremely high, highlighting the critical role of clinical suspicion and radiologist insight in facilitating accurate diagnoses [15]. Regardless of whether the ultimate pathology confirms benign SPL or contains a malignant component, it is important for radiologists to be aware of this post-procedural complication, as it mimics more common malignant processes such as peritoneal carcinomatosis [16]. Therefore, the purpose of this study is to describe the clinical and radiologic imaging features of SPL, with or without a malignant component, in women with a history of uterine leiomyoma intervention, such as myomectomy or hysterectomy.

## Methods

This retrospective HIPAA-compliant study was approved by the involved institutional review boards. The need for informed consent was waived. Multi-institutional review was performed at five centers to search via picture archiving and communication system (PACS) for any CT or other cross-sectional imaging cases with pathology-proven or suspected peritoneal leiomyomatosis. 31 cases of pathology-proven or suspected peritoneal leiomyomatosis were identified, including 28 cases after an intrauterine leiomyoma intervention (i.e., SPL) with index CT dates ranging from 2014 to 2024. The electronic medical record (EMR) was then searched for demographics, clinical history, surgical history, and pathology results. The PACS was further searched for all available imaging before index leiomyoma intervention, after intervention, and after debulking/resection intervention if performed (US, CT, MR, and/or PET/CT). Retrospective imaging review was performed independently by board-certified abdominal radiologists using predefined imaging variables; any discrepancies were resolved by.

Relevant findings from the index CT examinations, which were performed after the primary leiomyoma intervention, were recorded, including largest peritoneal-based mass, number of peritoneal masses (unless confluent or too numerous to count, TNTC), peritoneal location of masses, and imaging characteristics of the masses. Ultrasound images were reviewed for sonographic characteristics including margins, echogenicity, shadowing, and Doppler flow. MR imaging was reviewed for T1/T2 signal intensity and enhancement. Fluorine-18 fluorodeoxyglucose (FDG) PET/CT imaging was reviewed for FDG avidity. Cases were also reviewed for other findings, such as organ invasion, lymphadenopathy, and ascites.

Additional clinical details were obtained regarding presenting symptoms, type of initial leiomyoma surgery (including whether a morcellator and/or containment system was used), years from primary surgery and SPL diagnosis, treatment details (medical versus surgical), pathology, whether recurrence of symptoms or leiomyomas occurred, and total length of patient follow-up available.

## Results

The multi-institutional review yielded 28 female patients (mean age at index CT, 42.7 ± 10.6 years) with prior primary uterine leiomyoma intervention who developed SPL (with or without a malignant component).

### Primary uterine leiomyoma intervention

Pre-procedural pelvic imaging and/or radiology reports were available in 11/28 cases, including 5 US, 3 CT, and 3 MR examinations. Typical imaging characteristics of uterine leiomyomata were observed at US, CT, and MR prior to intervention. Mean size of the largest leiomyoma was 9.0 ± 4.3 cm (*n* = 10), and the mean number of lesions was 1.4 ± 0.8 (*n* = 7), with 3 cases reporting “multiple uterine leiomyomas”. The mean age of patients at the time of primary leiomyoma intervention was 36.6 ± 10.3 years (range, 17–71 years), with the primary intervention date ranging from years 2007–2019. Hysterectomy was performed in 16 of the patients (57.1%) and myomectomy was performed in 12 of the patients (42.9%). The majority of patients had a morcellator used during surgery (61.0%, *n* = 17); 5 patients did not have a morcellator used (18.0%, *n* = 5) and the remaining 6 cases were unknown (21.0%, *n* = 6). Whether a tissue containment system was used was unknown in 14 cases, used in 2 cases, and not used in one case. Example notes regarding the details of surgery in these cases included: “had containment system leak”, “had spillage during removal”, and “complex case with adhesions”. Where available, pathology from the primary uterine interventions confirmed benign leiomyomas (64.3%, *n* = 18). Pathology reports were not available in the remaining cases (35.7%, *n* = 10) regarding the primary leiomyoma intervention. Malignant pathology (leiomyosarcoma) was not documented in any of the primary leiomyoma resection cases when pathology was available.

### Post primary leiomyoma intervention (SPL diagnosis)

The demographics, clinical, and imaging findings of the 28 SPL cases are demonstrated in Table 1. Most patients were experiencing symptoms at the time of the index CT (78.6%, *n* = 22). The most common symptom was abdominal pain (60.7%, *n* = 17). Other symptoms included abdominal bloating (10.7%, *n* = 3), abnormal uterine bleeding (3.6%, *n* = 1), and urinary frequency (3.6%, *n* = 1). On physical exam, 7 of the patients (25.0%) were documented to have a palpable abdominal mass.

**Table 1:** Demographics, clinical, and imaging findings in 28 patients with SPL

Case	Age (index imaging)	Initial intervention	Years from primary intervention	Symptoms	Index CT: # lesions	Index CT: largest mass (cm)	Main lesion location	MR	US	Debulking surgery	Most aggressive pathology	Recurrence	Total follow up (years)
1	33	Hysterectomy	3.5	Yes	2	11.2	Pelvic	Yes	No	Yes	Leiomyomas	Yes	1
2	34	Hysterectomy	6	Yes	9	8.7	Pelvic (& trocar site)	Yes	Yes	Yes	Leiomyomas	Yes	2
3	44	Hysterectomy	1	Yes	1	5.5	Porta hepatis	Yes	Yes	Yes	Leiomyoma	No	0.2
4	37	Hysterectomy	5.8	Yes	6	7.2	Pelvic	No	Yes	Yes	Leiomyomas	No	5
5	49	Hysterectomy	5.7	Yes	4	18.9	Pelvic	No	Yes	Yes	Leiomyosarcoma	No	1
6	41	Myomectomy	10	Yes	6	3.5	Pelvic (& trocar sites)	Yes	No	Yes	Leiomyomas	No	13
7	57	Hysterectomy	9.3	Yes	5	2.4	Pelvic (& trocar site)	Yes	Yes	Yes	Leiomyomas	Unknown	No f/u
8	54	Hysterectomy	2	Yes	1	9.5	Pelvic	No	Yes	Yes	Leiomyoma	Unknown	No f/u
9	38	Hysterectomy	7	Yes	2	10	Pelvic	Yes	No	Yes	Leiomyomas	No	7
10	45	Hysterectomy	12	Yes	4	21	Pelvic	Yes	No	Yes	Leiomyomas	Unknown	No f/u
11	32	Myomectomy	8	Yes	12	1.6	Pelvic	Yes	No	No	N/A	No	1
12	36	Myomectomy	3	No	7	3.2	Pelvic	No	No	Yes	Leiomyosarcoma	Yes	11
13	57	Myomectomy	21	No	TNTC	1.1	Pelvic	No	No	Yes	Leiomyomas	No	4
14	42	Hysterectomy	2	No	2	0.8	Pelvic	Yes	No	No	Leiomyoma	No	4
15	41	Hysterectomy	5	Yes	5	7.5	Pelvic & perisplenic	No	Yes	Yes	Leiomyomas	No	1
16	35	Myomectomy	5	No	4	3.8	Pelvic	Yes	Yes	Yes	Leiomyomas	No	4
17	43	Myomectomy	11	Yes	14	8.7	Pelvic (& trocar site)	No	Yes	Yes	Leiomyomas	No	7
18	49	Hysterectomy	9	Yes	1	9.4	Pelvic (& trocar site)	Yes	No	Yes	Leiomyomas	No	1
19	47	Myomectomy	11	Yes	4	4.2	Pelvic (& trocar site)	No	No	Yes	Leiomyomas	No	1
20	46	Hysterectomy	8	Yes	5	15.5	Pelvic & RUQ	Yes	Yes	Yes	Leiomyomas	No	10
21	37	Myomectomy	13	No	2	8	Pelvic & RUQ	Yes	Yes	Yes	Leiomyomas	No	2
22	23	Myomectomy	7	Yes	2	4.8	Pelvic	No	Yes	Yes	Leiomyomas	No	5
23	29	Myomectomy	2	Yes	TNTC	20.6	Pelvic	No	No	Yes	Leiomyomas	Unknown	No f/u
24	48	Myomectomy	13	Yes	1	12.2	Pelvic & perihepatic	Yes	Yes	Yes	Leiomyosarcoma	Yes	4
25	54	Hysterectomy	10	Yes	1	5.9	Pelvic	Yes	No	Yes	Leiomyoma	No	3
26	35	Myomectomy	6	Yes	3	9.1	Pelvic	Yes	Yes	Yes	Leiomyomas	Yes	17
27	47	Hysterectomy	3	Yes	3	3	Pelvic	No	Yes	No	N/A	No	5
28	72	Hysterectomy	1	No	1	6.2	Pelvic	Yes	Yes	No	N/A	No	10

*TNTC* too numerous to count, *RUQ* right upper quadrant

**Fig. 1:**
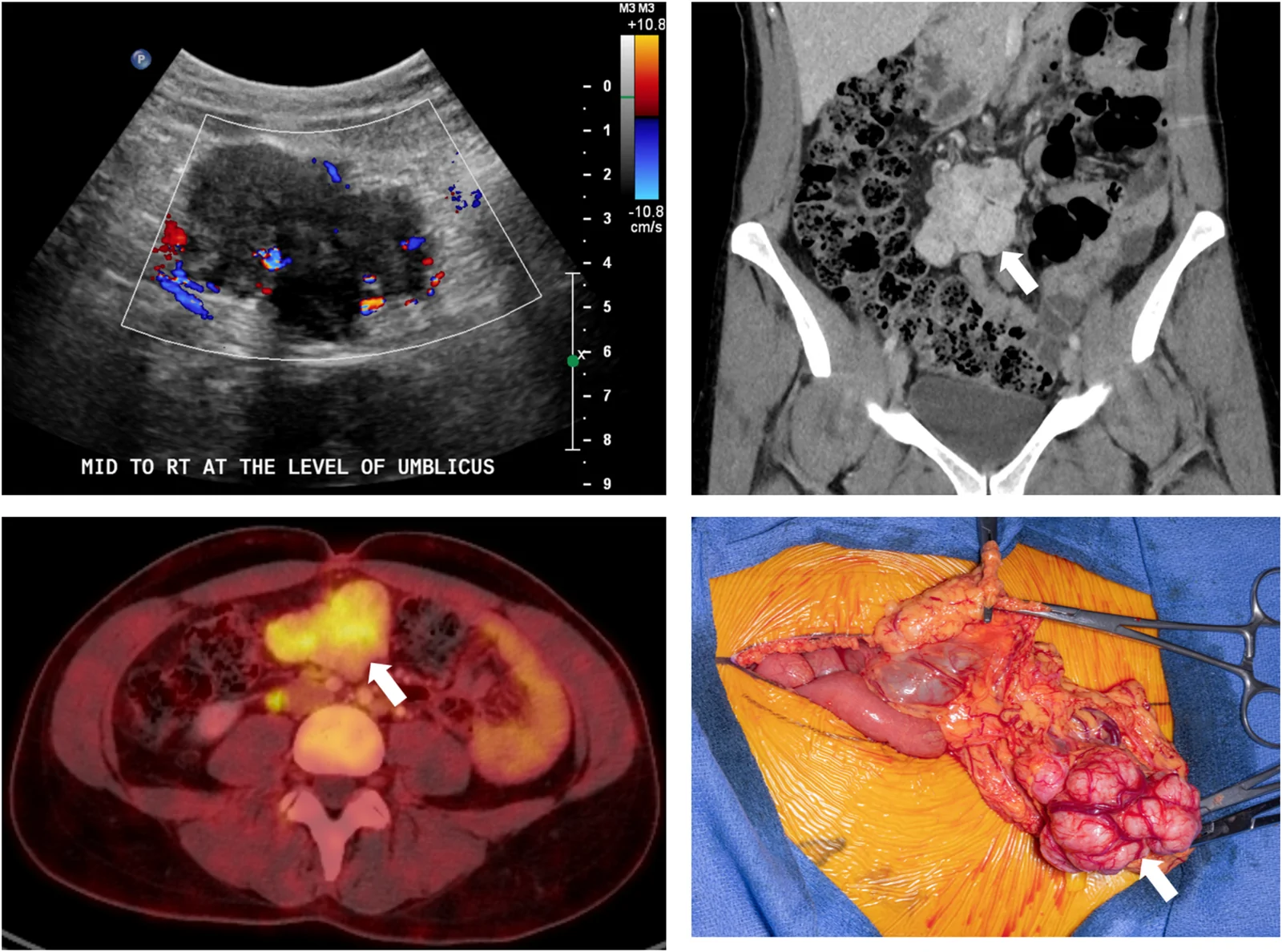
41-year-old female with history of robotic hysterectomy with morcellation of 11 cm fundal leiomyoma 5 years prior, presents with dysuria and pelvic pain (Case 15). Pelvic ultrasound with color Doppler (upper left) demonstrates a lobulated shadowing vascular lesion at the level of the umbilicus. Coronal CT of the abdomen and pelvis with IV contrast (upper right) redemonstrates a lobulated heterogenous pelvic mass (arrow). Fused FDG PET/CT image (lower left) demonstrates the lesion to be FDG-avid (arrow). Intraoperative image (lower right) from debulking resection with gross pathology of the lobulated pelvic mass (arrow). Final pathology resulted as leiomyoma without a malignant component

**Fig. 2:**
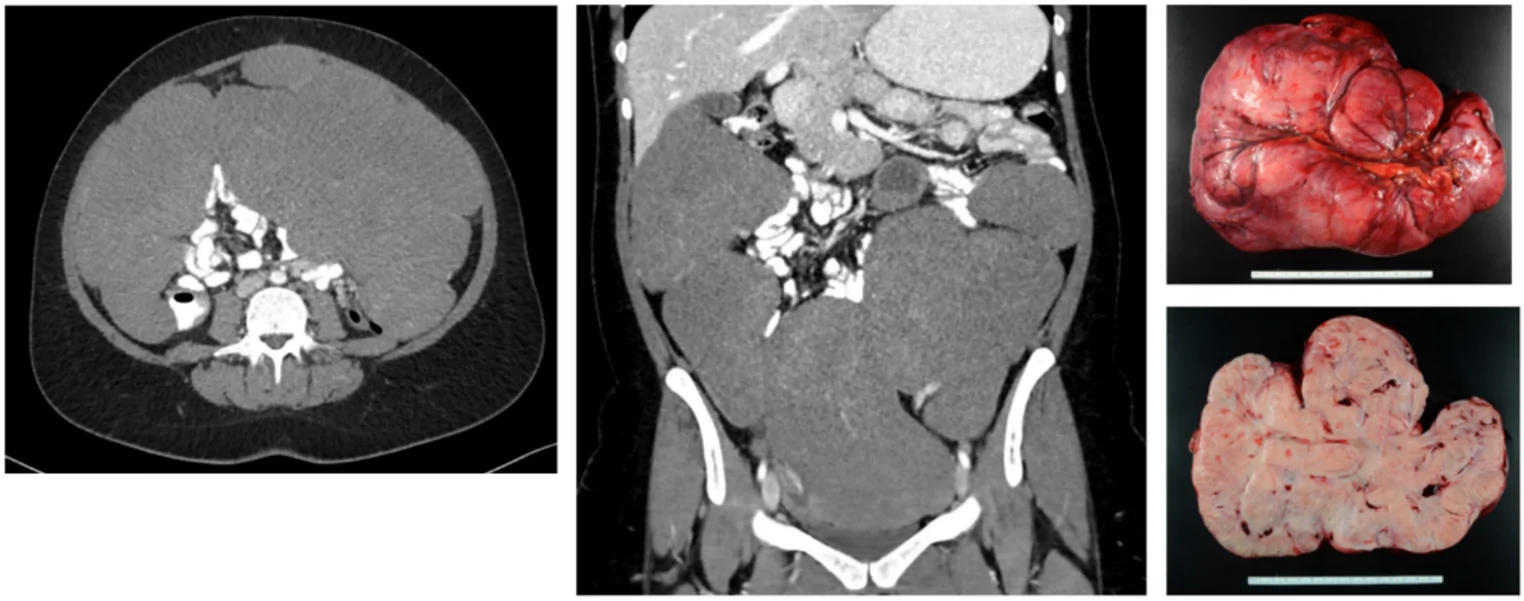
29-year-old female with history of large leiomyomas status post myomectomy performed during cesarean section 2 years prior, presents with abdominal pain and increased girth (Case 23). Axial (left) and coronal (middle) contrast-enhanced CT images demonstrate multiple large well-circumscribed homogeneous soft tissue masses throughout the peritoneal cavity. Patient underwent excision of abdominal masses with pathology returning as peritoneal leiomyomatosis without a malignant component. Gross pathology (upper right) demonstrates a well-circumscribed lobulated peritoneal mass and cross-section pathology (lower right) shows the classic “whorled” appearance of tightly packed bundles of smooth muscle

**Fig. 3:**
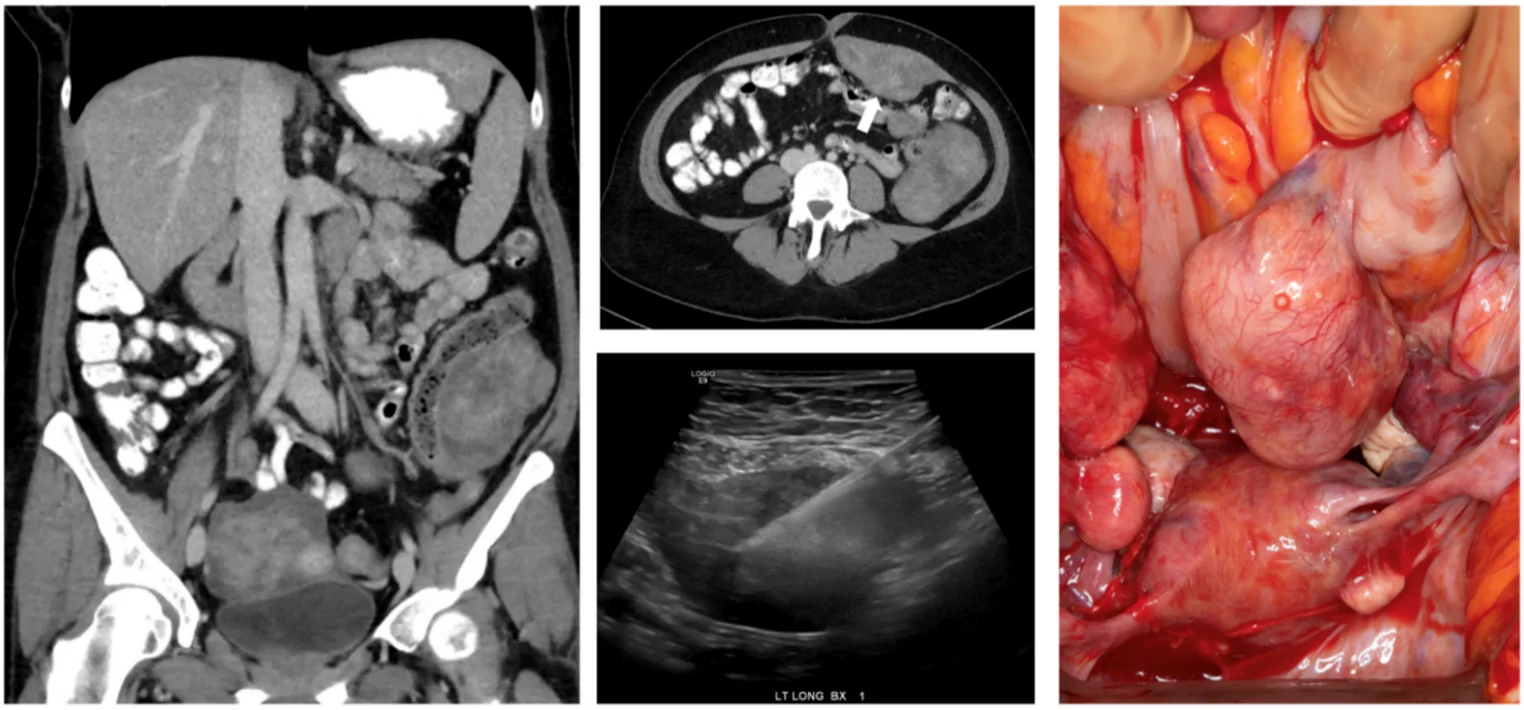
43-year-old female with history of three large uterine leiomyomas status post laparoscopic myomectomy with power morcellator 11 years prior, presents with weight loss and palpable mass within the left abdominal wall (Case 17). Coronal (left) and axial (upper middle) contrast-enhanced CT images demonstrate multiple circumscribed heterogenous lobulated masses throughout the abdomen, including one at the prior laparoscopic surgery trocar site in the left rectus muscle (arrow). Image from ultrasound-guided percutaneous biopsy (lower middle) was performed of the left lower abdominal wall mass, with pathology resulting as leiomyoma. The patient underwent debulking surgery due to symptoms. Intraoperative image (right) shows multiple lobulated peritoneal-based masses, consistent with biopsy proven SPL without a malignant component

**Fig. 4:**
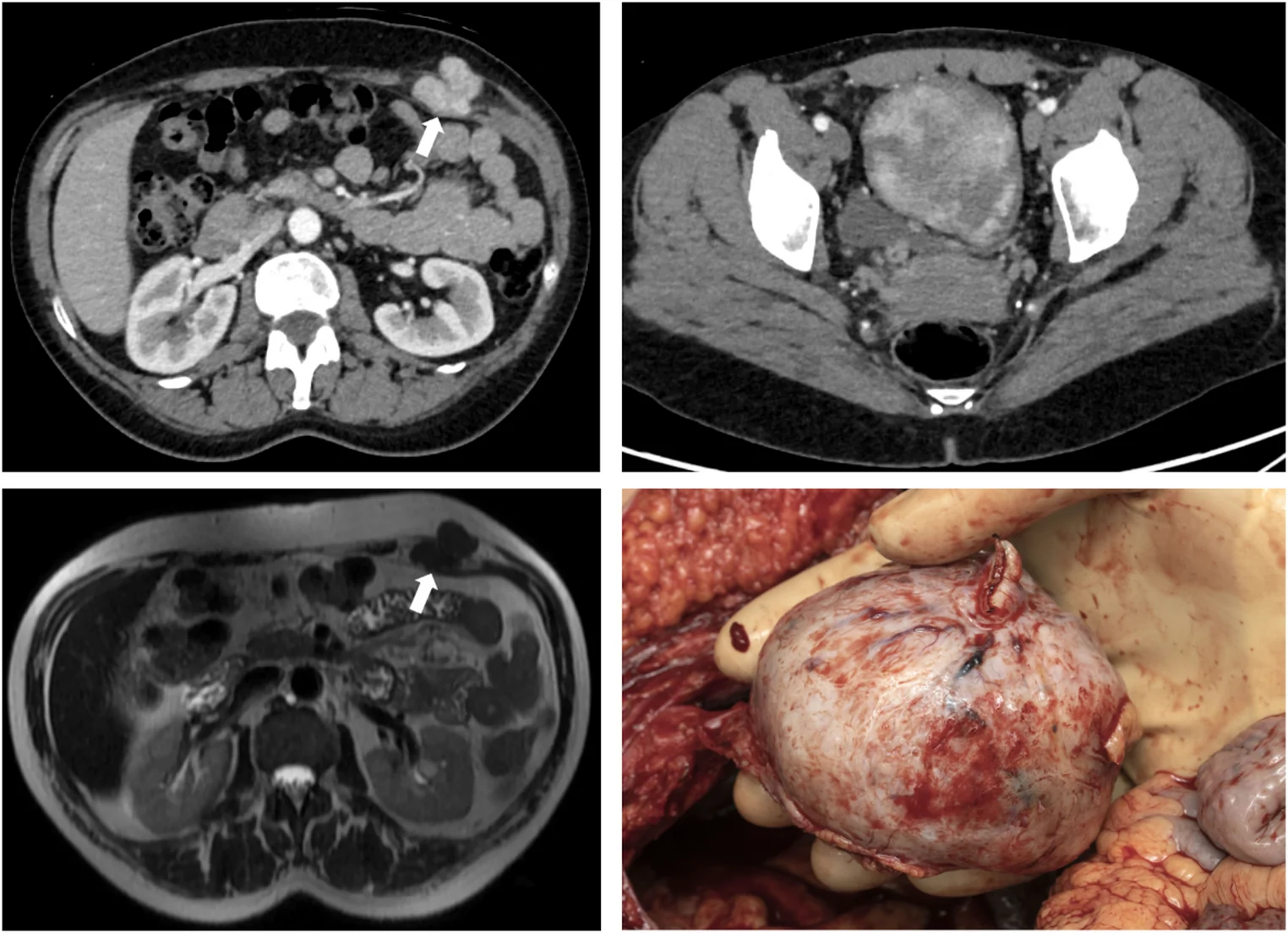
49-year-old female with history of laparoscopic hysterectomy with morcellation of uterine leiomyomas and reported spillage of some contents within the intraperitoneal cavity. Patient presents with pelvic pain and urinary frequency (Case 18). Axial contrast-enhanced CT image through the upper abdomen (upper left) demonstrates a lobulated enhancing mass at a prior laparoscopic surgery trocar site at the left rectus abdominis muscle (arrow). Axial CT image through the pelvis (upper right) shows another large intraperitoneal leiomyoma. Axial T2-weighted MR through the upper abdomen (lower left) redemonstrates the lesion at the trocar site (arrow). The patient underwent debulking surgery due to symptoms. Intraoperative image (lower right) shows a circumscribed peritoneal-based mass with final pathology of disseminated leiomyomatosis without a malignant component

**Fig. 5:**
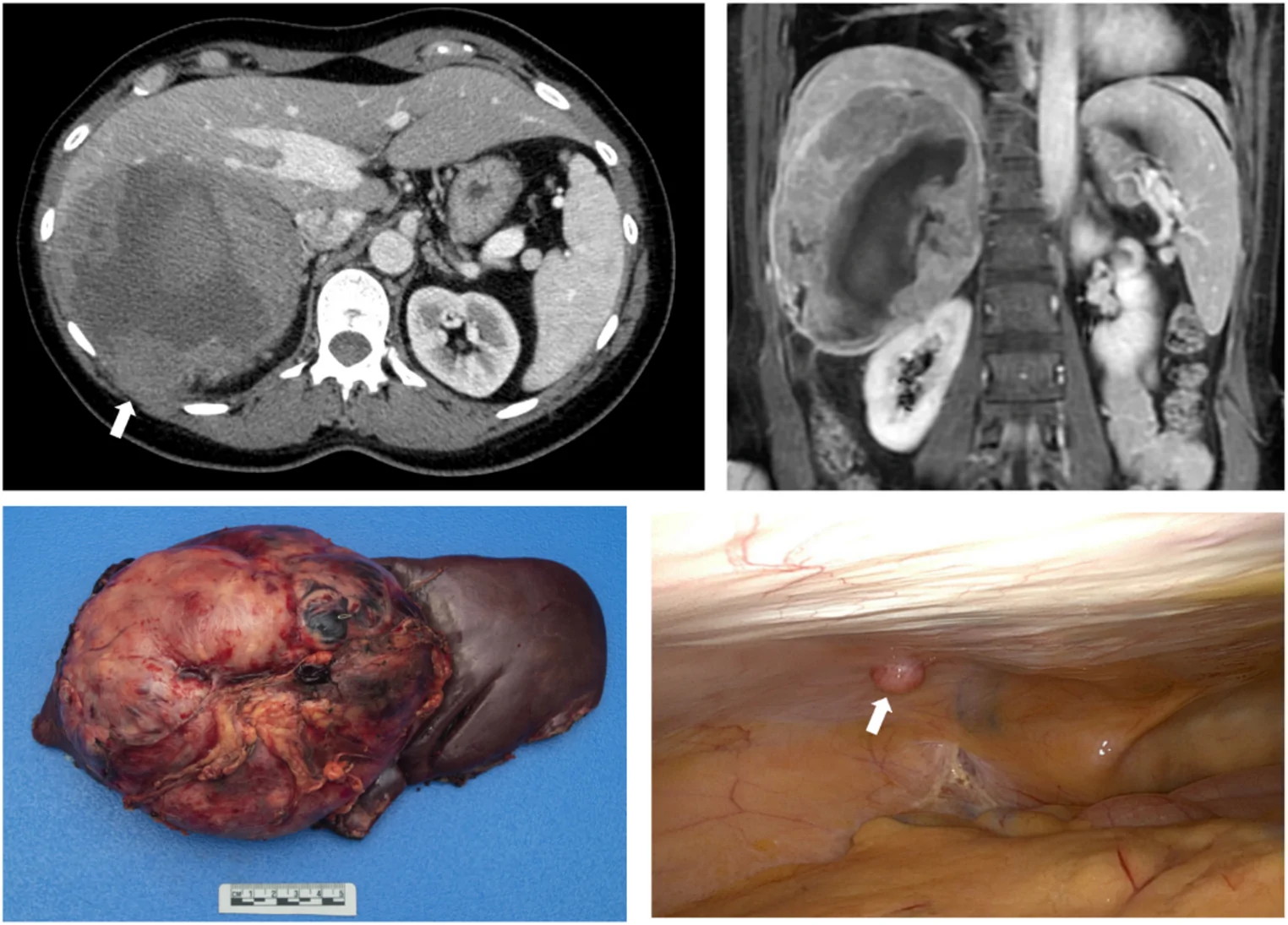
48-year-old female with history of laparoscopic myomectomy with morcellation of uterine leiomyomas 13 years prior, presents with persistent right upper quadrant pain (Case 24). Axial contrast-enhanced CT image of the upper abdomen (upper left) demonstrates a large heterogeneous perihepatic mass with an irregular infiltrating margin posteriorly (arrow). Coronal fat-suppressed contrast-enhanced T1-weighted MR image (upper right) re-demonstrates the large heterogeneous perihepatic mass with central necrosis. Patient underwent surgical resection of the mass and partial hepatectomy. Gross pathology image (lower left) of the aggressive infiltrating perihepatic mass with attached normal liver. Final pathology returned as high-grade leiomyosarcoma. Eight months later, the patient underwent an additional laparoscopic resection due to recurrence within the left anterior abdominal wall. Intraoperative laparoscopic image (lower right) demonstrates an abdominal wall recurrence (arrow)

The index CT diagnosing SPL was performed a mean 7.2 ± 4.5 years after the primary uterine leiomyoma surgical intervention (median, 6.5 years; range, 1–21 years). All 28 cases had a post primary leiomyoma intervention CT performed, 17 cases also had MR performed, and an additional 16 had ultrasound completed. Typical US/CT/MR findings included multiple rounded or lobulated, solid enhancing peritoneal-based nodules or masses with similar imaging characteristics to uterine leiomyomata (Figs. 1, 2, 3, 4 and 5). Additionally, two cases underwent FDG PET/CT for further evaluation, one of which demonstrated abnormal FDG avidity (Fig. 1) while the other did not show increased uptake. Excluding the two cases where the peritoneal-based lesions were confluent and/or TNTC, the mean number of peritoneal-based nodules or masses was 4.1 (range, 1–14). The mean size of the largest peritoneal mass measured 8.0 ± 5.6 cm (median, 7.4 cm; range, 0.8–21.0 cm). All but one case had at least one peritoneal-based mass present within the pelvis. Six cases had lesions at the prior laparoscopic surgery trocar sites (Figs. 3 and 4). Five cases had more atypical lesion locations including perisplenic, perihepatic, and porta hepatis (Fig. 5).

Percutaneous image-guided biopsy was performed in 11 cases prior to debulking surgery, with pathology returning as benign leiomyoma in nine cases, and nondiagnostic in two cases. Three of the cases were ultimately found to have a malignant component of leiomyosarcoma on pathology. In one case, a malignant dominant infiltrative and heterogeneous mass demonstrated adjacent hepatic and abdominal wall invasion with trace ascites (Fig. 5). The second case showed retroperitoneal lymphadenopathy, and the third case did not have imaging findings concerning for malignancy.

### SPL treatment, pathology, and recurrence

Nearly all the SPL cases underwent a debulking surgery (85.7%, *n* = 24). Of those patients, the pathology returned as benign leiomyomas in 21 of the cases (87.5%). As noted above, pathology in three of the cases returned with at least a focus of leiomyosarcoma (12.5%). One additional patient underwent medical treatment with Lupron for her CT guided biopsy-proven leiomyoma. Lastly, the three patients without pathologic diagnosis have been undergoing imaging surveillance for their presumed SPL, with two cases stable on imaging after 1.2 and 5 years, and another case that has been slightly increasing in disease burden over the past 10 years.

Out of our 24 cases who underwent debulking surgery, 5 were found to have documented SPL recurrence. Most of the lesions were present within the pelvis, although one patient had a left para-aortic lesion. No disease recurrence was found in 19 patients, and 4 patients were lost to follow-up. The average total follow-up interval from index CT for our cases was 4.2 years (range, 0.2–10.8 years).

## Discussion

Leiomyomatosis peritonealis disseminata (LPD), also known as disseminated peritoneal leiomyomatosis (DPL), was first described in 1952 by Willson and Peale and is characterized by the disseminated growth of extra-uterine leiomyomas throughout the peritoneum [4, 17]. These extra-uterine leiomyomas present as multiple peritoneal-based soft tissue nodules or masses, typically ranging from a few millimeters to several centimeters in size [16]. The precise underlying etiology remains unknown, although the main hypothesis for primary peritoneal leiomyomatosis is smooth muscle metaplasia within the submesothelial mesenchymal cells of the peritoneal lining resulting from transformation under hormonal stimulation [18, 19]. For our cases of SPL, however, a secondary iatrogenic etiology is present. Specifically, the use of power morcellators during surgery for uterine leiomyomas increases the risk of myomatous fragments retained in the peritoneal cavity and along the laparoscopic port tracts [19, 20]. A study completed by Cucinella et al. evaluated 423 women who had a myomectomy using a morcellator and found that 1.2% of the women developed pathology-proven secondary peritoneal leiomyomatosis over a 3-year period with all the cases having benign pathology [10]. Although less common, SPL can also occur after hysterectomy or myomectomy without associated morcellator use. Furthermore, Batton et al. even identified a rare case of SPL after uterine artery embolization [21].

In recent decades, there have been growing concerns regarding morcellation-related iatrogenic injuries, specifically when using an unconfined morcellation system. Interestingly, the primary focus centered around the risk for cancer spread (i.e., leiomyosarcoma), largely overlooking the more common complication of benign SPL. The United States Food and Drug Administration (FDA) issued a black box warning for the risk of cancer spread when using laparoscopic power morcellation in November 2014 [6]. After the warning, it was noted that the overall number of laparoscopic myomectomies and hysterectomies had subsequently decreased, with an associated increase in post-surgical complications and patient readmissions related to the rise of open surgical procedures [9]. Then, in December 2020, the FDA released an updated Safety Communication, which recommended only using a laparoscopic power morcellator with a tissue containment system to prevent the spread of undetected cancer cells and only in appropriately selected patients [22]. Again, the focus was mainly on rare cases of cancer and not the much more frequent SPL complication, which remains under-recognized. Additionally, if morcellation is performed within the abdominal cavity, it was recommended that the surgeon repeatedly apply liberal peritoneal irrigation after the procedure to ensure no myoma fragments remain [17].

In our study, we found that the US, CT, and MR imaging characteristics of SPL masses were overall similar to uterine leiomyomas, as expected, although only 11 patients had available pre-operative imaging. As pre-operative MR is increasingly used to risk-stratify leiomyomas [23], future studies of SPL may benefit from evaluating signal characteristics and morphologic behavior of leiomyomas prior to intervention, including intravenous extension. There was a wide variation in the number of peritoneal lesions per case, including six cases having a solitary secondary leiomyoma and two cases having diffuse “too numerous to count” disease extent. Excluding those two TNTC cases, the mean number of peritoneal-based masses was 4.1, with median of 3.5. In addition to peritoneal-based disease, deposits along the prior laparoscopic surgery trocar sites were also relatively common, seen in six cases in our series. Although data was limited to two patients, FDG PET/CT was found not to offer additional diagnostic utility for SPL, with one case demonstrating lesion avidity while the other did not. In the literature, the avidity of benign leiomyomatosis is also uncertain as uterine fibroids in general have been shown to demonstrate mild-moderate FDG avidity depending on the phase of menstrual cycle and cellularity, although it has also been reported that any intense uptake is concerning for sarcomatous transformation of the lesions [4, 24].

Beyond the commonly encountered entity of peritoneal carcinomatosis, the differential diagnosis for peritoneal-based soft tissue implants is broad and includes a wide array of other neoplastic and non-neoplastic conditions [25–28]. To help narrow the differential on CT or other cross-sectional imaging, it is critical for the radiologist to incorporate any relevant additional clinical or imaging information. In the case of SPL, this would primarily include recognizing a history of prior uterine fibroid intervention, as well as excluding any history of prior malignancy. Although both the medical literature and lay press have largely focused on the risk of peritoneal dissemination of leiomyosarcoma after fibroid morcellation [11–14, 16, 29], our findings support that benign SPL is a much more common complication. Previous literature estimates the occurrence of unsuspected uterine sarcoma in cases with leiomyoma morcellation to be between 1/278 and 1/1960 [30]. In our case series, three patients (12.0%) aged 36, 48, and 49, were found to have pathology-proven leiomyosarcoma after their SPL debulking surgery. For most patients presenting with peritoneal-based masses related to prior leiomyoma intervention, the pathology will be benign (nearly 90% in our series). These patients are also at risk for undue anxiety related to the focus on malignancy in the available literature.

Definitive management of SPL is unclear, with no established guidelines on treatment [31]. Patients typically undergo image-guided percutaneous biopsy or surgical resection for diagnosis. In our review, 11 cases had percutaneous biopsies (via US and/or CT) prior to surgical intervention. Currently, the mainstay of treatment is surgical excision (as with 89.3% of our cases), although hormonal blockade has been used as an alternative therapy (as with one of our cases) [32]. Thus, a multi-disciplinary approach is recommended as many of these patients may experience disease recurrence (as with 17.9% of our cases). As such, more research is warranted.

Data regarding benign SPL is more limited than the even more rare complication of peritoneal leiomyosarcoma. Nonetheless, prior studies focusing on benign SPL have found similar pathologic findings, although the majority of the patients were reportedly asymptomatic, unlike our series. A single site study by Chen et al. in 2021 found 13 cases with iatrogenic SPL following laparoscopic myomectomy with uncontained morcellation. They found an average time of presentation was 6.1 years after the initial surgery and that most of the patients were not symptomatic [7]. Another single-site study by Li et al. found 11 patients with pathologically-proven SPL with surgical history related to uterine leiomyomas (including 4 patients with mechanical morcellation). Most of these patients were asymptomatic with the size range of lesions from 0.5 to 25 cm (average size, 2–3 cm) [33].

There are several limitations to our study as this was a retrospective review of a relatively rare entity. Thus, there is limited pre-operative imaging, surgical reports and available pathology of the patient’s primary uterine leiomyoma intervention, mostly due to the procedure being performed at another institution and/or the substantial time interval between the initial intervention and subsequent diagnosis of SPL. Also, encountered bias may be present if only patients who were symptomatic were imaged. Additionally, as this was a multi-institutional study, variable imaging protocols were used. Three of our cases were presumed to be SPL based on clinical history and imaging findings and did not have proven pathology. Also, four of our cases were lost to follow-up. Further research could also be performed to assess the potential impact of the 2020 FDA Safety Communication regarding contained morcellation interventions.

This study describes a rare, mostly benign complication that can occur following surgical treatment for uterine leiomyomas. Although the literature states that the majority of these patients are asymptomatic, over 70% were symptomatic in our series. Importantly, only a small minority of cases (~10%) proved to be malignant. In summary, radiologists should be familiar with the imaging features and clinical presentation of secondary peritoneal leiomyomatosis, which can mimic malignancy and peritoneal carcinomatosis. Despite being a largely benign condition, most women in our multi-institutional review were symptomatic and underwent additional tumor debulking surgical intervention.

## Data Availability

No datasets were generated or analysed during the current study.

## Abbreviations

SPL: Secondary peritoneal leiomyomatosis
LPD: Leiomyomatosis peritonealis disseminate
DPL: Disseminated peritoneal leiomyomatosis
